# Albumin as an Effective Auxiliary Agent for the Enriched Extraction of Anthraquinones and Curcumin from Plant Matrices

**DOI:** 10.3390/molecules30020249

**Published:** 2025-01-10

**Authors:** Chiara Collevecchio, Salvatore Genovese, Francesco Epifano, Lorenzo Marchetti, Serena Fiorito

**Affiliations:** Department of Pharmacy, University “Gabriele d’Annunzio” Chieti-Pescara, Via dei Vestini 31, 66100 Chieti Scalo, CH, Italy; chiara.collevecchio@unich.it (C.C.); lorenzo.marchetti@unich.it (L.M.); serena.fiorito@unich.it (S.F.)

**Keywords:** albumin, anthraquinones, curcumin, emodin, extraction

## Abstract

Nowadays, several processes to enrich desired bioactive compounds in plant extracts have been developed. The objective of the present study was to assess the performance of bovine serum albumin in increasing the extractive yields of anthraquinones and diarylheptanoids from their respective raw plant powder extracts. Aloe emodin, rhein, emodin, and chrysophanol, from *Polygonum cuspidatum*, *Senna alexandrina*, *Rhamnus frangula*, and *Rheum palmatum*, and curcumin from *Curcuma longa* were analyzed in parent dry extracts, solubilized either with water, ethanol, or hydro-alcoholic mixtures, and in ones treated with aqueous solutions of bovine serum albumin by HPLC with UV/Vis detection. The different ratios between the volumes of solvents, powdered plant extracts, and bovine serum albumin were tested. The addition of albumin provided an increase in the yields of aloe emodin in the range 7.8–50.4-fold; of rhein in the range 6.1–14.1-fold; of emodin in the range 19.7–39.7-fold; of chrysophanol in the range 15.1–28.7-fold; and, finally, of curcumin of 32.1-fold. The addition of bovine serum albumin in the processing of plant extracts has been shown to be a novel and a valid alternative, comparing favourably to already reported methodologies. The easy-to-handle procedures, readily accessible facilities, and the employment of cheap substrates and reagents represent the most evident advantages of the methodology described herein.

## 1. Introduction

**Figure 1:**
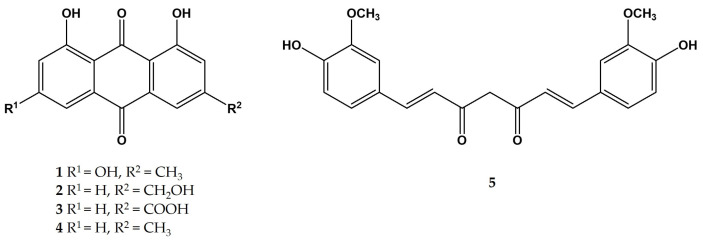
Structures of emodin **1**, aloe emodine **2**, rhein **3**, and chrysophanol **4**, and curcumin **5**.

The development of effective and powerful extraction methods is of pivotal importance for the optimal processing of phytopreparations. Indeed, modern techniques aim at maximizing the extraction yields of certain active ingredients from plant matrices. Furthermore, the use of nontoxic solvents and reagents for this purpose, in the context of a green chemical approach, and the reduction in operational times represent the main objectives in phytochemical research. Many methods have been developed to enrich bioactive compounds in plant extracts. These comprise liquid–liquid extraction; solid phase adsorption employing a wide panel of differently structured sorbent materials; high-speed counter-current chromatography; microwave-assisted, ultrasound-assisted, and pulsed electric field-assisted processes; deep eutectic solvents; pressurized, subcritical, and supercritical fluids; and several other techniques. All these methodologies have been excellently reviewed in recent years [1,2,3,4]. However, some of the listed methods suffer from several limitations, such as a low capacity, low extractive yields, and the need for expensive equipment. Thus, searching for novel, alternative, and more efficient plant extraction processes to maximize overall yields in selected active principles is still an intriguing and challenging field of research. In fact, improved methodologies play and will play an important role in providing consumers with high-quality herbal products both qualitatively and quantitatively. In this context, anthraquinones and diarylheptanoids, better known as “curcuminoids”, are among the most valuable phytochemicals extracted from medicinal, healthy, and food plants. Hundreds of phytopreparations, containing such secondary metabolites and claiming to exert beneficial effects on human health, are marketed all over the world. Numerous review articles summarizing the preventive and/or therapeutic effects of anthraquinones and curcuminoids have been reported in the recent literature [5,6,7,8,9,10]. Anthraquinones are found in a wide array of plant families, such as *Polygonaceae*, *Leguminosae*, *Rubiaceae*, *Rhamnaceae*, *Scrophulariaceae*, *Liliaceae*, *Verbenaceae*, and *Valerianaceae* and in few lower-order plants, like lichens [11]. Curcumin and curcuminoids have been extracted from *Curcuma longa* L. (turmeric) and other plants belonging to the family of Zingiberaceae [12]. A plethora of extraction methodologies for both classes of secondary metabolites, ranging from conventional techniques (e.g., Soxhlet extraction, maceration, and similar solvent extraction) to advanced technologies (e.g., ultrasound-assisted, microwave-assisted, enzyme-assisted, and supercritical fluid-promoted extraction) have been reported in the literature and several review articles focused on this topic [13,14]. The ideal solvent for plant extraction is water, but the limited solubility of the majority of biologically active components in vegetable matrices limits its effectiveness. Nevertheless, the use of organic solvents, with the notable exception of ethanol, is no longer sustainable, due to their toxicity, their residues in final products, and their high polluting power. In this context, we wish to report herein the use of bovine serum albumin (BSA) as an auxiliary and an adjuvant agent for the enriched extraction of both anthraquinones and diarylheptanoids from raw plant powder extracts. The use of this protein increased the yields of reference anthraquinones (namely, emodin **1**, aloe emodine **2**, rhein **3**, and chrysophanol **4**) and curcumin **5** (Figure 1).

Furthermore, BSA is readily commercially available at a relatively low cost, is easy to handle from a chemical point of view, and has good solubility in water. Natural and/or synthetic polymers (e.g., chitosans and molecularly imprinted polymers) have already been used in phytochemical practice for the effective extraction of several classes of plant secondary metabolites [15,16,17]. However, their costs are often very high, they require complex and money- and time-consuming chemical synthesis, and are sometimes less soluble in water, less chemically stable, and not easy to handle. Finally, BSA has been seen to tightly interact with both anthraquinones and diarylheptanoids [18,19,20,21,22,23]. All experimental steps were very easy to accomplish in a relatively short period of time. To the best of our knowledge, the present investigation represents the first example in the literature of the use of BSA to promote the extraction of these two classes of secondary metabolites in good to high yields. The use of nontoxic and non-polluting solvents and reagents and cheap materials in general represents substantial elements of novelty and renders our methodology a valid alternative comparable to the techniques so far reported in the literature for the same purpose.

## 2. Results

**Figure 2:**
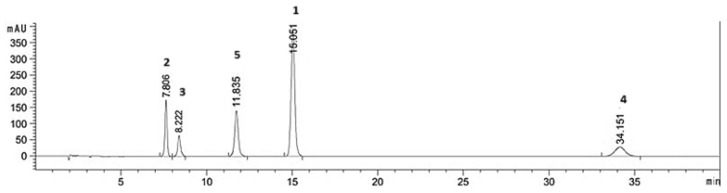
Representative HPLC chromatogram of the five secondary metabolites used as pure chemical standards.

In this paper, we investigated the capacity of BSA to increase the extraction yields of reference anthraquinones **1–4** and curcumin **5** in comparison to water and ethanol from plant powder extracts. We first established the calibration curves using a mixture of the five pure commercially available chemical standards and validated the HPLC method. The retention times recorded for the standard analytes were the following: aloe emodin **2** 7.8 ± 0.17 min., rhein **3** 8.2 ± 0.12 min., curcumin **5** 11.8 ± 0.14 min., emodin **1** 15.0 ± 0.15 min., and chrysophanol **4** 34.2 ± 0.11 min. A representative HPLC chromatogram is illustrated in Figure 2.

The subsequent experimental acquisition was derived from the treatment of the parent plant material with water and ethanol and from the related quantification of reference anthraquinones **1–4** curcumin **5** in solution. The results of the quantification are reported in Table 1.

The data reported in Table 1 clearly provides evidence of the poor solubility in H_2_O of practically all the secondary metabolites under investigation. The samples listed in Table 1 were evaporated to complete dryness to re-obtain a powder that was subsequently treated with solutions of BSA to evaluate its solubilizing effect on the five compounds under investigation. After centrifugation, the supernatants were directly injected into the HPLC apparatus, providing the data reported in Table 2.

The results reported in Table 2 clearly reveal a marked shift to higher values in the content of all of the reference phytochemicals in aqueous solutions, highlighting the effective capacity of BSA to promote the greater solubilization of the five refence compounds. In particular, for the concerned anthraquinones, the aloe emodin content in solution increased by 50.4-, 8.4-, and 7.8-fold in *P. cuspidatum*, *R. frangula*, and *R. palmatum*, respectively, while no substantial increases were recorded in *S. alexandrina*; the rhein content increased by 6.1- and 14.1-fold in *P. cuspidatum* and *R. palmatum*, respectively; the emodin content increased by 19.7- and 39.7-fold in *P. cuspidatum* and *R. frangula*, respectively; the chrysophanol content increased 28.7- and 15.1-fold in the case of *P. cuspidatum* and *R. palmatum*, respectively. Some anthraquinones, namely, rhein in *S. alexandrina*, emodin in *R. palmatum*, and chrysophanol in *R. frangula*, for which the quantities in the aqueous solutions of the parent extracts were below the LOQ, could be easily quantified after the addition of BSA. Finally, the curcumin content increased by 32.1-fold in the case of *C. longa*. The above recorded effects may be explained by the capacity of BSA to form complexes with anthraquinone and diarylheptanoid skeletons [24,25,26,27,28,29]. To carry out further experiments to gain more insights into the performance of BSA, *P. cuspidatum* and *C. longa*, as the plant sources, and emodin and curcumin, as the sample phytochemicals, were selected. Thus, to assess the dose-dependence profile on the extractive yields of compounds **1** and **5**, increasing amounts of the protein, namely, 20 mg, 50 mg, 80 mg, 100 mg, and 200 mg, were added to the aqueous solutions of the respective parent plant powders. The results of this experimental step are reported in Table 3.

**Figure 3:**
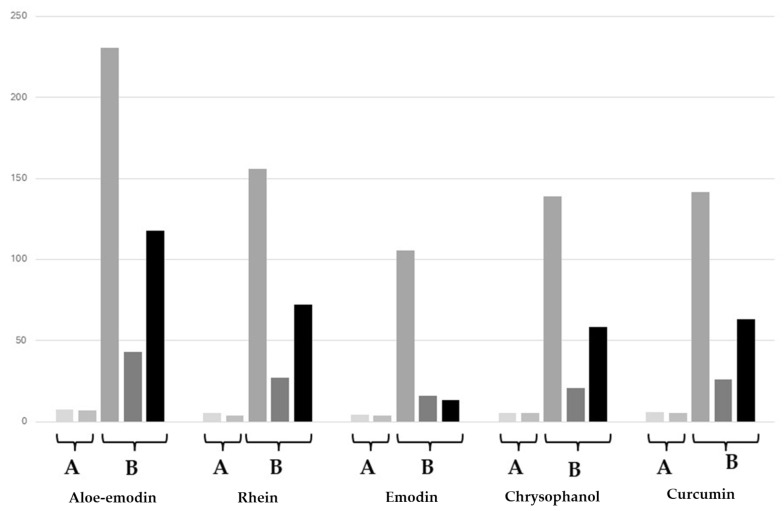
Content of aloe emodin, rhein, emodin, chrysophanol, and curcumin in the parent plant powders of *P. cuspidatum* and *C. longa* after treatment with methods A (bars on the left) and B (bars on the right).

The data reported in Table 3 show how using quantities < 100 mg of BSA did not result in any improvements in the yield of curcumin from *C. longa,* while doubling the amount of the protein practically led to no increases. On the other hand, a marked dose-dependent pattern for the increase in yield was recorded for curcumin. In this case, it may be hypothesized that an excessive dilution of BSA may prevent an efficient interaction between the protein and the secondary metabolite of interest. A strong interaction may also be hampered by the presence in the plant extract of other components, like polysaccharides [30,31]. Consequently, we set up two further experiments with the aim of using more concentrated solutions of BSA. The first method, named method A, was accomplished by first dissolving the two parent plant powders in 0.625 mL of ethanol, then by slowly adding to the resulting mixture a 1% *w*/*v* aqueous solution of BSA. A second method, named method B, was developed by preparing similar hydro-alcoholic solutions, with fixed amounts of parent plant powders and the addition of 1% *w*/*v* of BSA (prepared with 30 mg, 60 mg, and 100 mg of BSA), and then subjecting them to freeze-drying. The resulting solid dispersions were then resuspended into 10 mL of water and injected into the HPLC apparatus for quantification. The content in the anthraquinone and curcumin samples were, in both cases, analyzed by HPLC in the solutions not treated and treated with BSA in the same way as reported above. The indicated volume of EtOH has been optimized in terms of maximizing the yield in the selected secondary metabolites. Table 4 and Figure 3 show the results of the related quantifications.

Table 4 clearly shows that, following method A, the contents in the five reference compounds were close to that recorded for the ethanolic extracts and already reported in Table 2. Since the presence of ethanol, in which the sample phytochemicals are soluble, could affect the results of this assay, additional experiments were performed by reducing its volume to 0.350 mL. The results in Table 4 confirmed this hypothesis, since there is a direct decrease in the content of anthraquinones and curcumin in the corresponding extracts. The addition of increased amounts of BSA resulted in a significant increase in the anthraquinone solubility in water in a dose-dependent manner: aloe emodin **1** and emodin **3** showed the highest enhancement, of around 82-fold and 61-fold, respectively, while the content of curcumin increases by around 6.2-fold. Moreover, in the case of curcumin, the addition of ethanol and BSA solution seems to be a very good combination in terms of yields, especially when considering that the solubilization with only ethanol provided a value of 25.04 ± 0.12 mg.

In all cases, the experiments were performed at room temperature and neutral pH as the optimal and validated operating conditions. Even though we did not investigate further the possible effects of both these chemico-physical parameters on the formation of complexes between BSA and anthraquinones and curcumin, we found that changing both the temperature (up to 70 °C) and pH (down to 1 or up to 12) did not provide better results than those reported in Table 2, Table 3 and Table 4 in terms of the times and yields. On the contrary, in some instances (higher temperatures and pH > 10), the extensive chemical degradation of the target analytes was recorded.

## 3. Discussion

Wishing to adopt water as the extractive solvent, the ideal remedy to overcome the very poor solubility of the majority of active principles from plants is to use auxiliary agents and adjuvants to increase the capacity of such compounds to dissolve in water and thus to increase the extractive yields. In particular, in the case of naturally occurring anthraquinones, some relevant methodologies to this aim have been reported in the recent literature. In 2015, Li and coworkers, after careful investigation of its dissociation constants and treating plant extracts with diluted aqueous solutions of NaOH, succeeded in solubilizing emodin in water as a sodium salt [31]. The most common auxiliary extractive agents reported in the literature for anthraquinones are represented by cyclodextrins [32], chitosans [33], ionic liquids [34], and chemically different-structured hydrogels [35]. Much more has been reported in the literature about methodologies to increase the water solubility of diarylheptanoids, represented in most cases by curcumin. Numerous effective techniques are available nowadays, and these include encapsulation in nanoparticles and nanotubes, the use of cyclodextrins, the crystallization of the desired compound in polymorphic forms, and the formation of cocrystals. All these methodologies have been recently and explicatively reviewed [36]. However, some of the listed processes may have some drawbacks like difficulties in synthesizing the adjuvant materials for extraction, low extractive yields, and poor selectivity towards classes of plant secondary metabolites. Albumin, in general, either human, bovine, egg, and/or from other sources has found limited usage in phytochemical practice. In most cases, this protein has been the main component of supramolecular structures (e.g., nanoparticles and nanotubes) used only at a later time for extraction purposes [37,38,39,40]. In other cases, BSA was added to the reaction medium as a buffering agent [41]. In only a few cases, BSA has been employed as an auxiliary agent for the extraction of selected compounds from plant matrices. In particular, this protein has been used to promote the adsorption of cellulose from root extracts of *Glycyrrhiza glabra* [42], to facilitate the extraction of lignophenols [43], and to increase the water solubility of the same curcumin [44]. This scarce use of albumins in phytochemical practice is quite surprising, considering that there are several pioneering studies describing, in great detail, the surface properties of these proteins and suggesting how their interaction with albumins leads to the substantial stabilization of numerous chemically labile (e.g., oxidation to air) active principles from plant extracts [45,46,47]. Concerning anthraquinones and diarylheptanoids, spectroscopic and docking studies have revealed how a tight interaction between these secondary metabolites and albumins can be achieved in the solution state [18,19,20,21,22,23]. Such findings represent the theoretical and practical basis of our investigation and are responsible for the observed enrichment of these selected phytochemicals from the respective plant sources. The data we described in the present investigation confirm the tight interactions that can be easily and rapidly achieved between anthraquinones and curcuminoids and BSA in the solution state, leading to a great increase in the extractive yields of molecules that are typically insoluble in water. Even if a large amount of BSA vs. the quantity of raw extract (100 mg vs. 10 mg) has been used, the advantage of recovering significantly larger quantities of target analytes compared to traditional extraction methods compensates for the use of a relatively high amount of BSA. In turn, this finding allowed us to obtain, by conventional extraction processes, the same secondary metabolites in largely greater yields.

## 4. Materials and Methods

### 4.1. Chemistry and Plant Materials

Emodin (purity ≥ 99%, HPLC), aloe emodin (purity ≥ 98.5%, HPLC), chrysophanol (purity ≥ 99%, HPLC), rhein (purity ≥ 99%, HPLC), and curcumin (purity ≥  97.5%, HPLC) were purchased from Extrasynthese (Genay, Lyon Cedex, France) and used as HPLC chemical standards without further purification. Bovine serum albumin (BSA) (lyophilized powder, purity ≥ 96%, agarose gel electrophoresis) was provided by Merck Sigma Aldrich (Darmstadt, Germany). Double-distilled H_2_O (HPLC-grade, resistivity > 18.2 MΩ cm) used to carry out all extractions and HPLC analysis was obtained by treatment with an Elix 3 and Milli-Q purification system (Millipore, Bedford, MA, USA). EtOH (purity ≥ 99.8%, GC), MeOH Chromasolv^®^ (purity ≥ 99.9%, GC), and HCOOH (purity 98–100%, acidimetric) were purchased from Merck Sigma-Aldrich (Darmstadt, Germany). CH_3_CN (purity ≥ 99.9%, GC) was provided by Fisher Scientific Italia (Rodano, Milan, Italy). Stock solutions of the four standard anthraquinones and curcumin were individually prepared at a concentration of 1.0 mg/mL by dissolving 10 mg of each reference powder into a 10 mL volumetric flask with MeOH. The stock solutions were finally collected into one flask and then subjected to HPLC analysis for the elaboration of calibration curves. Solid dry raw extracts of *Polygonum cuspidatum* (roots, titrated at 50%, emodin), *Senna alexandrina* (leaves), *Rhamnus frangula*, *Rheum palmatum* (rhizomes), and *Curcuma longa* (rhizome, titrated at 65%, curcumin) were purchased from local markets and authenticated by the authors. Voucher specimens (PC-DE-2020-1), (SA-2020-1), (RF-2020-1), (RP-2020-1), and (CL-2020-1) have been stored in the repository of the laboratory of Chemistry of Natural Compounds and Phytochemistry of the Department of Pharmacy, University “G. D’Annunzio” of Chieti-Pescara. Aqueous extracts were obtained by suspending 10 mg of each of the dry raw plant material mentioned above in 10 mL of H_2_O and leaving the suspensions under magnetic stirring for 5 hs at room temperature (rt). After filtration under vacuum, the resulting solutions were used both for the HPLC analysis of the parent extracts and for ones obtained after treatment with BSA. Ethanolic extracts were prepared by dissolving 2.5 mg of the powdered plant material in 10.4 mL EtOH and then directly subjecting into HPLC.

### 4.2. Evaluation of the Solubilizing and Stabilizing Effects of BSA

To evaluate BSA’s capacity to enhance the otherwise poor solubility of the target analytes in H_2_O, 100 mg of the protein was added to 5 aliquots of 10 mL of H_2_O and kept under magnetic stirring at rt for few minutes. Subsequently, 10 mg of the dry raw extract was added and each of the resulting suspensions was kept under vigorous magnetic stirring for 5 h at rt. After centrifugation (5000 rpm, 5 min, rt), the supernatants were directly injected into the HPLC apparatus. The solubilizing effect on emodin was also tested by adding different amounts of BSA (in the range 20–200 mg) to *P. cuspidatum* aqueous extract. To give further insight on BSA’s effectiveness on *P. cuspidatum* and *C. longa* extracts, two more processes, named method A and B, were investigated. The first procedure was carried out by dissolving 10 mg of the two powdered extracts in 0.625 mL of EtOH each, followed by the dropwise addition of a 1% *w*/*v* aqueous solution of BSA (9.375 mL). The resulting suspension was left under magnetic stirring at rt for 30 min., centrifuged as described above. The supernatant was subsequently directly injected into the HPLC apparatus. Since the presence of EtOH in the hydro-alcoholic solutions could affect the solubility of the reference phytochemicals, additional assays were performed by reducing the amount of EtOH used to dissolve the extracts to 0.35 mL. On the other hand, method B was accomplished by dissolving 10 mg of each of the two extracts in three aliquots of 0.500 mL of EtOH, followed by the dropwise addition of different volumes of a 1% *w*/*v* aqueous solution of BSA prepared using increasing amounts (100 mg, 60 mg, and 30 mg) of BSA to reach a final volume of 10 mL. The resulting hydro-alcoholic solutions were freeze-dried for 30 h using a Lyovapor L-200 apparatus (Büchi Labortechnik AG, Flawil, Switzerland). The resulting fine powders were then resuspended in 10 mL of H_2_O, the resulting suspension being maintained under vigorous magnetic stirring for 10 min. and centrifuged as described above. The supernatant was subsequently injected into the HPLC apparatus [24]. Standard samples, prepared by dissolving 10 mg of each dry raw extracts in 10 mL of EtOH, were injected into HPLC and used as a reference for the evaluation of the solubility.

### 4.3. HPLC-DAD Analyses and Method Validation

HPLC analyses were carried out using an Agilent 1100 (Santa Clara, CA, USA) series instrument equipped with an autosampler, a binary solvent pump, and a diode-array (DAD) detector. For the four anthraquinones and curcumin, separation was achieved employing a Kromasil^®^ RP C18 (4.6 mm ∅ × 150 mm, 5 µm particle size, Nouryon, Goteborg, Sweden). The mobile phase consisted of a 99.6: 0.4 H_2_O: HCOOH mixture as solvent A and a 99.6: 0.4 CH_3_CN: HCOOH mixture as solvent B, operating in gradient mode at a flow rate of 1.0 mL/min. The gradient system was applied as the following: 0.0–3.0 min. 2–40% B; 3.01–5.0 min. 40% B; 5.01–38.0 min. 50% B; 38.01–40.0 min. 50–2% B. The column temperature was set at 25 °C and the injection volume was 20 µL. The wavelength for both qualitative and quantitative analyses were set at 254 nm for the anthraquinones and at 435 nm for curcumin. Calibration curves were drawn by injecting standard solutions of a mixture of aloe emodin, rhein, curcumin, emodin, and chrysophanol at 9 concentration values, namely, 0.5 μg/mL, 1.0 μg/mL, 2.5 μg/mL, 5.0 μg/mL, 10.0 μg/mL, 15.0 μg/mL, 25.0 μg/mL, 50.0 μg/mL, and 100.0 μg/mL. The HPLC method was validated according to the ICH guidelines in terms of the following parameters: precision, accuracy, linearity, limits of detection (LOD), and limits of quantification (LOQ). These and other relevant data have been summarized in Table S1 of the Supplementary Materials. The intra-day and inter-day precisions were determined following the already reported procedure [16]. Precision was measured at three concentration levels for quality control (QC) samples, namely, QC_Low_ = 0.5 μg/mL, QC_Medium_ = 10.0 μg/mL, and QC_High_ = 100.0 μg/mL. Accuracy was calculated by spiking samples derived from the extraction of vegetable materials with three concentrations of the pure chemical standards mixture (low, medium, and high spikes). LOD and LOQ values were determined by the injection of serial dilutions of the corresponding standard solutions, featured by a signal-to-noise (S/N) ratio of 3.3 and 10.0 as the reference, respectively. All calculations were performed in triplicate.

### 4.4. Statistical Analysis

Statistical analyses were elaborated following the same general procedure, namely, Student’s *t* test, as already reported in the literature [25].

## 5. Conclusions

The addition of BSA to extractive media consisting of only water and/or hydro-alcoholic mixtures, with the aim of further processing plant extracts, has been shown to be a novel and a valid alternative methodology that favourably compares to the already existing techniques in the same field. The process we described herein allowed us to obtain, in very good yields, biologically active selected anthraquinones and curcuminoids. Such yields were significantly higher than those recorded by using only water as the extractive solvent and, in some cases, when using only ethanol, a solvent in which both anthraquinones and diaryheptanoids are soluble. The easy-to-handle procedures, readily accessible facilities, relatively short operational times, especially when considering HPLC analyses, and the employment of cheap substrates and reagents represent the most evident advantages of the methodology described herein. The easiness and versatility of the methodology we described herein could be quickly transferred to the extraction of other classes of secondary metabolites, like flavonoids and polyphenols in general, towards which albumins showed a great affinity [48]. Such experiments are now in due course in our laboratory.

## Figures and Tables

**Table 1:** Quantification of aloe emodin, rhein, emodin, chrysophanol, and curcumin in aqueous and ethanolic solutions obtained after treatment of the respective parent plant powder extracts (values are expressed as mg/g ± SD of dry extract).

	H_2_O				
	Aloe Emodin	Rhein	Emodin	Chrysophanol	Curcumin
*P. cuspidatum*	0.07 ± 0.003	0.43 ± 0.02	2.30 ± 0.12	0.48 ± 0.13	ND
*S. alexandrina*	0.08 ± 0.002	ND	ND	ND	ND
*R. frangula*	0.12 ± 0.002	ND	0.27 ± 0.06	ND	ND
*R. palmatum*	0.09 ± 0.001	0.15 ± 0.05	ND	0.17 ± 0.06	ND
*C. longa*	ND	ND	ND	ND	0.32 ± 0.04
	**EtOH**				
	**Aloe Emodin**	**Rhein**	**Emodin**	**Chrysophanol**	**Curcumin**
*P. cuspidatum*	7.72 ± 0.23	6.62 ± 0.31	204.54 ± 0.62	47.71 ± 0.25	ND
*S. alexandrina*	0.40 ± 0.03	1.65 ± 0.06	ND	0.16 ± 0.002	ND
*R. frangula*	2.34 ± 0.02	ND	14.80 ± 0.08	1.25 ± 0.01	ND
*R. palmatum*	1.37 ± 0.11	3.39 ± 0.23	0.85 ± 0.09	3.93 ± 0.02	ND
*C. longa*	ND	ND	ND	ND	134.94 ± 0.16

ND = not detected or below LOQ.

**Table 2:** Quantification of aloe emodin, rhein, emodin, chrysophanol, and curcumin in the aqueous solutions of plant extracts obtained after evaporation to complete dryness of water solutions of parent powder extracts and after addition of BSA (10 mg/mL) (values are expressed as mg/g ± SD of dry extract).

	Aloe Emodin	Rhein	Emodin	Chrysophanol	Curcumin
*P. cuspidatum*	3.53 ± 0.08	2.64 ± 0.09	45.27 ± 0.08	13.77 ± 0.05	ND
*S. alexandrina*	0.08 ± 0.002	0.39 ± 0.01	ND	ND	ND
*R. frangula*	1.01 ± 0.01	ND	10.73 ± 0.12	0.89 ± 0.02	ND
*R. palmatum*	0.70 ± 0.01	2.12 ± 0.03	0.20 ± 0.01	2.57 ± 0.02	ND
*C. longa*	ND	ND	ND	ND	10.27 ± 0.04

ND = not detected or below LOQ.

**Table 3:** Quantification of emodin and curcumin in the aqueous solutions of parent plant powders with increasing amount of BSA (values are expressed as mg/g ± SD of dry extract).

	BSA (mg)				
	20	50	80	100	200
Emodin (*P. cuspidatum*)	10.40 ± 0.07	21.05 ± 0.03	39.41 ± 0.09	45.27 ± 0.08	45.89 ± 0.05
Curcumin (*C. longa*)	ND	ND	ND	10.27 ± 0.03	10.45 ± 0.04

ND = not detected.

**Table 4:** Quantification of aloe emodin, rhein, emodin, chrysophanol, and curcumin in the parent plant powders of *P. cuspidatum* and *C. longa* after treatment with methods A and B.

EtOH (mL)	Method A				
	Aloe Emodin	Rhein	Emodin	Chrysophanol	Curcumin
0.625	7.76 ± 0.15	7.01 ± 0.12	230.42 ± 0.84	43.28 ± 0.86	117.78 ± 0.96
0.350	5.37 ± 0.11	3.72 ± 0.08	155.72 ± 0.97	27.7 ± 0.54	72.34 ± 0.78
**BSA (mg)**	**Method B**				
	**Aloe Emodin**	**Rhein**	**Emodin**	**Chrysophanol**	**Curcumin**
30	4.3 ± 0.23	3.98 ± 0.19	105.36 ± 0.87	15.88 ± 0.67	13.65 ± 0.54
60	5.42 ± 0.54	5.34 ± 0.39	138.93 ± 0.95	20.56 ± 0.54	58.32 ± 0.46
100	5.72 ± 0.63	5.54 ± 0.44	141.37 ± 0.78	26.07 ± 0.35	63.20 ± 0.73

## Data Availability

The original contributions presented in this study are included in the article/Supplementary Material. Further inquiries can be directed to the corresponding authors.

## Supplementary Materials

The following supporting information can be downloaded at: https://www.mdpi.com/article/10.3390/molecules30020249/s1, Table S1. Calibration curves, precision, and accuracy data.
