# Supplementary material for: Albumin as an Effective Auxiliary Agent for the Enriched Extraction of Anthraquinones and Curcumin from Plant Matrices
Source: Molecules. 2025 Jan 10;30(2):249. doi: 10.3390/molecules30020249 (PMC11767785; doi:10.3390/molecules30020249)
Supplement: Supplementary file 1 [file molecules-30-00249-s001.zip › molecules-3381380-supplementary.pdf]

**Table S1.** Calibration curves, precision, and accuracy data.

| | Aloe-emodin | Rhein | Emodin | Chrysophanol | Curcumin |
|---|---|---|---|---|---|
| **Slope** | 3278 | 1351 | 3130 | 2491 | 2406 |
| **Intercept** | -214 | -423 | -464 | -397 | -413 |
| **$SD_s$ (slope)** | 562 | 212 | 491 | 334 | 109 |
| **$SD_i$ (intercept)** | 74 | 81 | 77 | 78 | 56 |
| **$r^2$** | 0.9997 | 0.9990 | 0.9992 | 0.9990 | 0.9999 |
| **LOD*** | 0.56 | 0.51 | 0.51 | 0.52 | 0.56 |
| **LOQ*** | 1.71 | 1.54 | 1.54 | 1.57 | 1.71 |
| **Precision (% RSD)** | | | | | |
| Intra-day** | | | | | |
| $QC_{low}$ | 100.2 / 0.998 | 100.1 / 0.999 | 100.3 / 0.999 | 100.1 / 0.996 | 100.0 / 0.995 |
| $QC_{medium}$ | 100.1 / 0.995 | 100.0 / 0.994 | 100.1 / 0.996 | 100.4 / 0.998 | 100.2 / 0.996 |
| $QC_{high}$ | 100.2 / 0.999 | 100.2 / 0.999 | 100.1 / 0.997 | 100.2 / 0.998 | 100.2 / 0.995 |
| Inter-day** | | | | | |
| $QC_{low}$ | 100.0 / 0.994 | 100.1 / 0.998 | 100.0 / 0.998 | 100.2 / 0.995 | 100.1 / 0.996 |
| $QC_{medium}$ | 100.1 / 0.996 | 100.1 / 0.999 | 100.2 / 0.998 | 100.4 / 0.998 | 100.2 / 0.998 |
| $QC_{high}$ | 100.3 / 0.998 | 100.2 / 0.999 | 100.1 / 0.996 | 100.3 / 0.999 | 100.1 / 0.994 |
| **Accuracy (% Recovery)** | | | | | |
| Intra-day** | | | | | |
| $QC_{low}$ | 100.7 / 0.992 | 100.8 / 0.999 | 100.9 / 0.994 | 101.1 / 0.997 | 100.5 / 0.994 |
| $QC_{medium}$ | 100.5 / 0.994 | 101.0 / 0.997 | 100.8 / 0.996 | 101.0 / 0.997 | 100.6 / 0.997 |
| $QC_{high}$ | 100.9 / 0.993 | 100.9 / 0.994 | 100.9 / 0.999 | 100.9 / 0.996 | 100.5 / 0.999 |
| Inter-day** | | | | | |
| $QC_{low}$ | 100.4 / 0.999 | 101.0 / 0.999 | 100.9 / 0.996 | 100.6 / 0.994 | 101.2 / 0.999 |
| $QC_{medium}$ | 100.7 / 0.998 | 100.9 / 0.997 | 100.5 / 0.998 | 100.6 / 0.997 | 101.0 / 0.997 |
| $QC_{high}$ | 100.9 / 0.998 | 100.9 / 0.997 | 100.6 / 0.999 | 100.9 / 0.998 | 100.9 / 0.997 |

*Values expressed as µg/mL; ** n = 6
